# Factors Influencing Proteolysis and Protein Utilization in the Intestine of Pigs: A Review

**DOI:** 10.3390/ani11123551

**Published:** 2021-12-14

**Authors:** Alina Kurz, Jana Seifert

**Affiliations:** 1 HoLMIR—Hohenheim Center for Livestock Microbiome Research, University of Hohenheim, 70599 Stuttgart, Germany; alina.renz@uni-hohenheim.de; 2 Institute of Animal Science, University of Hohenheim, Emil-Wolff-Str. 8, 70599 Stuttgart, Germany

**Keywords:** protein utilization, pigs, gastrointestinal tract, proteases, amino acids, transporter

## Abstract

**Simple Summary:**

In order to minimize protein requirements and nitrogen emissions in pig production, it is important to understand the physiology of protein breakdown in the pig’s gastrointestinal tract and to find ways to improve the protein utilization efficiency of the animals. In this review, we summarize and discuss factors influencing protein degradation and thereby affecting the efficiency of the animals. We shed light on the individual pillars of protein breakdown, starting with the enzymatic breakdown of the fed protein, via the amino acid transporters absorbing in the intestine, to the proteolytic capacity of the microbial and animal-specific proteome. The available literature shows the specific activities and expression levels of proteolytic enzymes and AA transporters depending on the availability of free or bound AA in the feed. Improvements in nutrient digestibility result from changes in nutrient transporter and enzyme gene expression, as well as a change in microbial activity. We are of the opinion that in the future, also with the help of transcriptomics, more time should be invested in researching the physiology of protein degradation and the associated protein utilization efficiency using highly standardized animal trials.

**Abstract:**

Pigs are among the most important farm animals for meat production worldwide. In order to meet the amino acid requirements of the animals, pigs rely on the regular intake of proteins and amino acids with their feed. Unfortunately, pigs excrete about two thirds of the used protein, and production of pork is currently associated with a high emission of nitrogen compounds resulting in negative impacts on the environment. Thus, improving protein efficiency in pigs is a central aim to decrease the usage of protein carriers in feed and to lower nitrogen emissions. This is necessary as the supply of plant protein sources is limited by the yield and the cultivable acreage for protein plants. Strategies to increase protein efficiency that go beyond the known feeding options have to be investigated considering the characteristics of the individual animals. This requires a deep understanding of the intestinal processes including enzymatic activities, capacities of amino acid transporters and the microbiome. This review provides an overview of these physiological factors and the respective analyses methods.

## 1. Introduction

The production of pork contributes to significant ecological damage through the generation of greenhouse gases and emissions of nitrogen compounds. Even if there are higher greenhouse gas emitters such as ruminants and specific industrial processes, we can have a positive influence on this damage by optimizing pork production [1,2]. In order to maintain body functions and the growth of the animal, the need for amino acids (AA) has to be met by the regular supply of proteins and free AA with the feed. As proteins consist of an average of 16% nitrogen (N) [3], large amounts of N compounds are produced, which are excreted in the feces and urine as degradation products of feed proteins. This is due to the problem that the precaecal digestibility of AA is currently in a range of 70 to 90% in the conventional protein feed components [4] and that specificities of single AAs have to be considered. For example, the Society for Nutritional Physiology indicates an intermediate utilization (63%) of the digestible portion of lysine for the protein approach [5]. These limits mean that the overall efficiency of protein utilization is currently barely higher than a third, which means that two thirds of the protein used is excreted by the animals [4]. However, not only nitrogen emissions, for example in the form of ammonia, lead to a negative ecological balance, but also the global production and trade of protein-rich feedstuff [6]. Additionally, limited acreage leads to a competition between feed and food production [7]. One approach to minimize the explained problems would be to improve the protein utilization efficiency of the pigs to achieve an equal amount of animal protein with less raw protein intake. This could reduce the use of protein-rich feed and simultaneously reduce the excretion of harmful nitrogen compounds [6,8]. A recent study showed that 30% of the animals fed a 20% crude protein-reduced diet showed a growth rate similar to that of the animals fed with the control diet and thus had a significantly higher protein efficiency than the average of the control group [9]. A reduction of the raw protein content in the ration of growing pigs with simultaneous AA supplementation was applied in another study and resulted in a reduced proportion of N in the urine and the total N excretion, without negatively affecting performance or carcass quality [10,11]. Besides the gross characterization of N incomes and outcomes, the physiological capacities and mechanisms to achieve the efficient protein utilization should be studied. This was rather neglected in the past, but it would be of great benefit to reveal more information about the physiological processes behind the N turnover in the animal body and to include this in future breeding strategies. The present review provides an overview of the different parameters that may be studied in order to gain better knowledge about the involved mechanisms. First, an overview of the most important proteases in pigs will be given, and the modern detection methods using kinetic and fluorescent reactions for enzymes will be explained. Second, we discuss the absorption of the produced AA in the intestine carried out by AA transporters and the respective detection methods. Third, we discuss the intestinal microbiome described as a possible key player in the protein turnover for the monogastric animals. High-resolution methods such as (meta-)proteomics can be used as a complementary approach to study the changes of the host and microbiome proteins depending on the applied feedstuff and provide additional information on their impact for an improved protein efficiency.

## 2. Proteolytic Enzymes in Pigs

### 2.1. Physiology and Biochemistry of Proteolytic Enzymes

The digestion of proteins begins in the stomach by the secretion of gastric juice, which consists of water, mucus, hydrochloric acid, pepsin and an intrinsic factor, a glycoprotein that forms complexes with vitamin B12 to ensure its absorption in the ileum. Pepsin is primarily responsible for the N-terminal cleavage of proteins into peptides and amino acids, thus making them accessible for the absorption in the small intestine. In the stomach, pepsin is released from the main cells in its inactive form, pepsinogen, to protect the stomach from self-digestion. The acidic environment in the stomach, which is generated by the hydrochloric acid, ensures that the pepsinogen is converted into pepsin [12].

**Figure 1:**
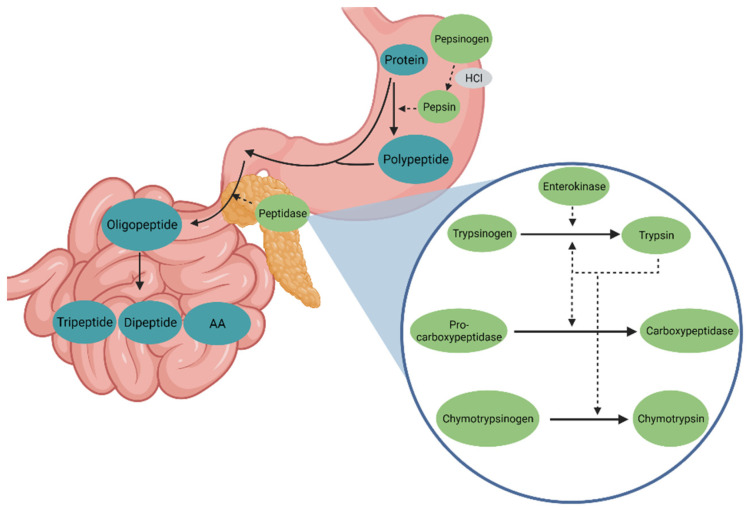
Peptidases in the stomach and small intestine and their activation after secretion from the pancreas.

In addition, feed proteins are known to be hydrolyzed in the small intestine by endo- and exopeptidases [13]. Activated proteolytic enzymes specifically cleave protein chains with a sequence-specific mode of action. The endopeptidase trypsin hydrolyzes peptide bonds between arginine and lysine and any other amino acid, whereas the endopeptidase chymotrypsin cleaves between leucine and methionine and, in the case of aromatic AA, between phenylalanine, tyrosine and tryptophan. The endopeptidase elastase hydrolyzes between alanine, valine, glycine, tyrosine, phenylalanine and leucine. Carboxypeptidases are exopeptidases and cleave phenylalanine, tyrosine, arginine and lysine residues at the carboxyl terminal end of the proteins [14,15,16,17,18]. The peptidases are secreted by the acinar cells of the pancreas in an inactive form by exocytosis, which serves to protect the surrounding tissue from self-digestion. Trypsinogen is secreted as the inactive form of trypsin, proelastase as the inactive form of elastase, chymotrypsinogen as the inactive form of chymotrypsin and procarboxypeptidase A and B as the inactive form of carboxypeptidase A and B. The secretion granules of the acinar cells contain a mixture of these enzymes. The pancreatic enzymes are only activated in the small intestine. In the case of peptidases, the enteropeptidase enzyme, which is localized in the brush border membrane of the proximal small intestine, acts as an endopeptidase and activates the trypsinogen to trypsin by splitting off a hexapeptide near the N-terminus of the zymogen and attend by conformational changes [14]. This process is also catalyzed by mold proteases. Trypsin also activates the remaining trypsinogen and the other peptidases by autocatalytic peptide cleavage [14]. Elastase is formed by trypsin activation of the zymogen proelastase. This step requires trypsin to cleave a peptide from the N-terminal end of proelastase what forms a new N-terminal amino group and allows the molecule to adopt its active form [19]. For chymotrypsin, the activation starts with trypsin splitting of the Arg15-Ile16 peptide bond. This leads to the formation of π-chymotrypsin. Further autocatalytic incidents produce δ-chymotrypsin, K-chymotrypsin and y-chymotrypsin as intermediate states of α-chymotrypsin [20]. In addition, with the digestive enzymes of the pancreas, a trypsin inhibitor is secreted, which prevents the activation of the trypsin in the pancreas. Figure 1 shows the peptidases in the stomach and the small intestine and their activation in the small intestine after secretion by the pancreas.

The relative abundance of peptidases expression can be studied by quantitative reverse transcription PCR (RT-qPCR) quantifying the mRNA level of the respective gene [21]. Together with the gene expression level of ß-actin, used as a housekeeping standard gene, the gene expression of various protease genes was analyzed to study the relative variation among different crude protein contents in pigs [17,18,22,23]. This enumeration is a good indicator for the potential enzyme activity in respect to different dietary treatments or other factors but it does not give any information on the true enzymatic activity. Thus, methods to study the proteolytic activity in intestinal samples were established and explained in more detail in the following chapter.

### 2.2. Detection of Enzymatic Activities

A common method to measure enzyme activities is the detection of the respective product or the consumption of a cofactor. Changes of absorption spectra induced by these two options are measured using a spectrophotometer [24]. In case of exo- and endopeptidases, the common methods are based on the detection of the formed product. The activity of pepsin is quantified by using a synthetic peptide substrate. Cleavage of this substrate releases a fluorophore-bearing peptide fragment and generates a fluorescence signal. The measurement takes place at a wavelength of 328 nm for excitation and 418 nm for emission [25]. Trypsin: in the commercially available detection kits for trypsin, a substrate is used to generate the chromophore p-nitroanilide (pNA), which can be measured at an absorbance of 405 nanometers (nm). This value is directly proportional to the trypsin activity in the sample [25,26]. In the case of chymotrypsin, a synthetically produced, fluorescent substrate is often used, which enables kinetic measurements of the chymotrypsin activity. Additionally, a supplied chymotrypsin activator leads to the conversion of chymotrypsinogen into the active form, which hydrolyzes the non-fluorescent substrate and causes stable fluorescence. The detection takes place kinetically with a wavelength of 380 nm for excitation and 460 nm for emission [25]. The elastase activity is also measured by detecting the fluorescence that occurs when the substrate is degraded. The measurement takes place at an emission maximum of 515 nm [25]. In the case of carboxypeptidases, no commercial kits are currently available. A protocol was recently published using N-(4-methoxyphenylazoformyl)-Phe-OH potassium salt and N-(4-methoxyphenylazoformyl)-Arg-OH HCl as substrates for carboxypeptidase A and B, respectively. Here, the kinetic absorbance measurement is also carried out in a spectrophotometer at 350 nm for 10 min [27]. Table 1 shows the different peptidases that are involved in protein digestion and their products after cleavage, modified from [17].

### 2.3. Factors Influencing Proteolytic Enzymes and Their Effects on Proteolysis

The pancreatic secretion in monogastric animals is strongly promoted by feed intake stimuli such as the low pH value in the duodenum and the protein and fat digestion products in the small intestine [17,18]. The pattern of enzymes synthesized and secreted by the pancreas adapts to the feed composition over a period of several days. For example, high-starch feeding leads to an increase in amylase in the pancreas, while high-fat feeding leads to an increase in lipases and protein-rich feeding to an increase in peptidases [17,18].

Since the composition of the secreted enzymes adapts to the feed composition, the influence of the ratio composition on the secreted enzymes in different organisms was examined in various studies [28,29,30,31]. The protein concentration in the small intestine, which reflects the protein concentration in the feed, influences the qualitative and quantitative secretion of pancreatic proteases. Some studies in rats showed that animals fed a low protein diet secreted less pancreatic trypsin and chymotrypsin than animals fed a high protein diet [28,29]. The proportion of raw protein and AA in the ration is likely to influence protein digestion and AA absorption in pigs. A study examined the trypsin and chymotrypsin activity, as well as the AA concentration in the blood serum of pigs that were fed either a low-protein diet, an AA-supplemented diet or a diet with a high protein content [32]. The results showed no significant interaction between ration and intestinal tract segment, but it was shown that the animals fed with a high protein content diet showed higher activities of trypsin and chymotrypsin than animals fed with other diets. This was especially true for trypsin in the jejunum compared to the duodenum. The authors assumed that the secretion of the proteases was influenced by the presence of pancreatic enzymes and their substrates in the small intestine without a reliable regulation mechanism [32]. It was also assumed that the presence of active trypsin, chymotrypsin and elastase in the small intestine blocks the secretion of the pancreatic juice whereas the proenzymes are unable to suppress the secretion [33,34]. In contrast, the infusion of trypsin inhibitor led to an enhanced secretion of proteases [35]. Fushiki et al. (1989) proposed that the secretion of pancreatic enzymes in rats and pigs is regulated by a negative feedback mechanism, which is caused by the activity of trypsin and chymotrypsin in the intestine [29]. Liddle et al. (1986) showed that proteins in the ration, which are susceptible to the activity of the proteases, stimulate the secretion of pancreatic enzymes by temporarily acting as a trypsin inhibitor [36]. This could explain why the lower levels of protein, arginine and lysine led to a reduced amount of trypsin and chymotrypsin in the pigs which were fed a low-protein diet [32]. A study with weaners, however, showed that trypsin activity was not affected by the reduced crude protein content (17% and 14%) of the ration [24]. The authors explained this by pointing to an improved nitrogen utilization of both feeding groups compared to the group with a high protein content (20%) [24]. Gene expression of the digestive enzymes was studied by He et al. (2016) and showed that growing pigs fed with a crude protein content of 12% in the diet had a lower mRNA level of trypsinogen, chymotrypsin B and chymotrypsin C compared to the animals with an 18% raw protein content in the ration [22]. A difference in gene expression between the 15% and the 18% raw protein content diet could not be determined. A decrease in the trypsinogen level was also evident during the final fattening phase in animals fed with 12% instead of 16% crude protein content in the diet. The lowest levels of trypsinogen gene expression were observed in the animals that had 10% crude protein in the ration. The proportion of expression levels of carboxypeptidase showed no difference in the animals of final fattening phase in any of the three groups. This indicated that a reduced gene expression for protein digestion in the intestine goes along with a reduced protein content in the ration [22].

The pancreatic enzymes have been extensively studied [28,29,30,31,37]. Studies in pigs have already shown that the activity of the digestive enzymes and their variations due to dietary changes determine how effectively dietary compounds influence the expression of these enzymes and therefore may promote weight gain [37].

Consistently, several working groups investigated the effect of different diets or supplements on the activity of the enzymes in the stomach and intestine [38,39,40,41,42,43]. In a study from Liu et al. (2020), the effect of spermine supplementation on the digestive abilities, the amino acid transporter expression and the metabolism in piglets was investigated. They showed that the piglets with spermine supplementation had an increased lipase and trypsin activity in the jejunum. This led to a higher digestibility of fat and protein in the jejunum. The spermine supplementation may improve digestion by increasing the abundance of digestive enzymes and expression of amino acid transporters [38]. Similar results were shown in the study from Fang et al. (2016) where they investigated an increase in sucrose and maltase in spermine supplemented pigs [39]. The effect of different protein sources as a replacement for fish meal in low protein diets on the intestinal digestive physiology was studied by Li, Hou [40]. They concluded that pigs fed the concentrated degossypolized cottonseed protein diet had higher concentration of pepsin in the stomach than those fed the other diets. Differences in the enzymatic activity in pancreas and jejunum could not be shown [40]. Yagami et al. (2017) researched the effect from a dietary rice- soybean meal-based diet as a substitute for a corn-soybean meal-based diet. They concluded that neither the activity of maltase, sucrose, aminopeptidase or dipeptidil peptidase IV in the duodenum differed significantly between the diets. In the jejunum, the activity of maltase and aminopeptidase was significantly higher in the rice-fed piglets. So far, the reasons for this effect are not clear and are being investigated further [41].

## 3. Amino Acid Transporter in Pig Intestine

### 3.1. Relevant Amino Acid Transporter in the Pig Intestine

**Figure 2:**
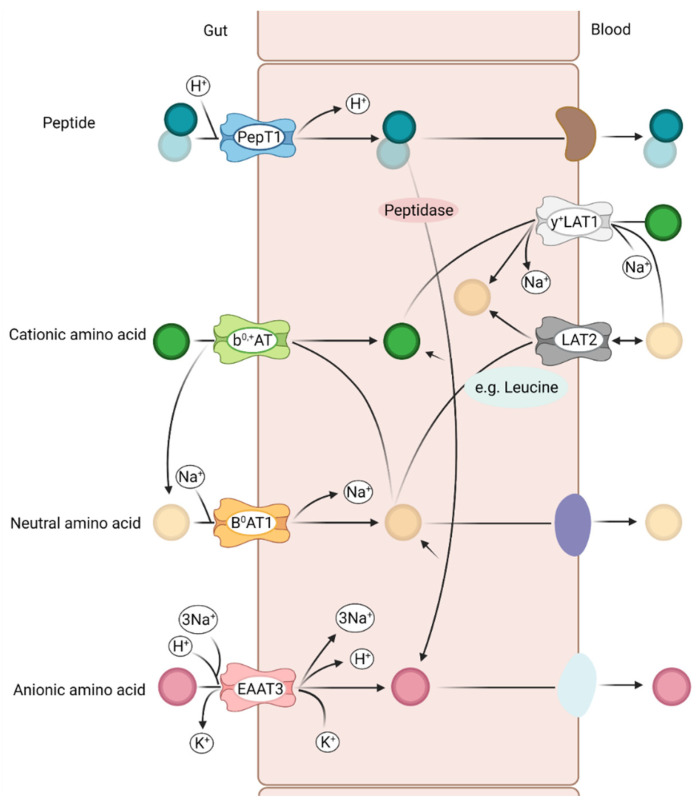
Peptide and amino acid transport systems in the intestine. The EAAT3 transporter is an important transporter for anionic amino acids. The Na^+^-coupled transporter B^0^AT_1_ is the major transporter for neutral amino acids. Additionally, b^0,+^AT transporter are located at the luminal membrane as the major transport system for cationic amino acids. Majorly acid antiporters such as LAT2 and y^+^LAT1 are expressed at the basolateral membrane, enabling a physiologically important amino acid replacement. PepT1 is an important transporter for intact peptides, which are not degraded into amino acids. Modified from Poncet and Taylor [46].

Numerous transporters with various substrate-specificities for a broad range of nutrients can be found in the intestine and some of these are solely responsible for the transport of AA. These include the SLC1, SLC6 and SLC7 transport systems, which are Na^+^/K^+^ ATPase transporters driven by established Na^+^ and H^+^ gradients in the epithelial basolateral membrane (Figure 2). Peptides are absorbed by H^+^-coupled symporters and are largely hydrolyzed to AA in epithelial cells, even if some peptides are brought into the bloodstream as intact peptides [44,45,46]. Neutral and anionic AA are absorbed by intestinal epithelial cells via a Na^+^-coupled symporter (cotransport), whereas cationic and double-basic AA are mostly transported by an AA antiporter, i.e., an exchange mechanism. Most essential AA are neutral AA and are absorbed apically by the neutral amino acid transporters B^0^AT_1_ (SLC6A19) and ASCT2 (SLC1A5) [46]. The b^0,+^AT can be found in the cationic AA transporter SLC7A9 and y^+^LAT1 can be found in the SLC7A7 transporter [47,48]. Individual and substrate-specific representatives can be found in the respective transport systems. For example, the transporter SLC1A1 transports glutamate, cysteine and aspartates, whereas the transporter SLC7A transports arginine, lysine, histidine and ornithine. All AA reach the bloodstream through an AA antiport or an efflux system from the epithelial cell. In particular, the glutamine and essential AA transport on the basolateral membrane offers the advantage of providing glutamine as a source of energy during the absorption of the essential AA into the bloodstream [46,49,50].

In addition, there are Na^+^-dependent amino acid carriers in the basolateral membrane, through which the epithelial cell is supplied with amino acids for protein synthesis and for energetic purposes if no amino acid is resorbed from the intestinal lumen. This applies, in particular, to the crypt cells and the large intestinal epithelium, since the brush border membrane of the cells does not have any Na^+^-dependent amino acid carrier [17,18].

The detection of amino acid transporters is commonly undertaken with RT-qPCR similar to the detection of proteases. Specific primers for the transporters were designed according to their published sequence in gene databases by several authors [32,51]. In addition, a housekeeping gene such as ß-actin is used, which can be inserted as an endogenous control to normalize the variations and to quantify the relative abundance of the transporter gene [52].

### 3.2. Influence of Amino Acid Transporter Expression and Their Effect on Protein Efficiency in Pigs

The level of freely available AA in the feed seems to influence the expression frequency of AA transporters in the small intestine of pigs [53]. This proposed association was studied by Morales et al. (2017) assuming that a lower protein content in the ration leads to a lower expression rate of AA transporters and that the reduced expression also has an effect on the serum concentration of these AA [32]. The activity of trypsin and chymotrypsin in the duodenum and jejunum, the expression of the two cationic AA transporters b^0,+^AT (SLC7A9) and y^+^LAT1 (SLC7A7) and a neutral AA transporter B^0^AT_1_ (SLC6A19) in the duodenum, jejunum and ileum as well as the concentration of free AA in the serum was analyzed. It could be shown that there is a significant connection between the dietary treatment and the intestinal segment, which is why the influence of the diet on gene expression in each intestinal segment was considered individually. The authors were able to show that the expression of mRNA for b^0,+^AT in the duodenum was higher in the animals that were fed with a low protein supplemented with AA diet (LPAA). In general, the expression level was higher in duodenum than in the jejunum. In the jejunum and ileum, no difference was found between the LPAA and high protein (HP) animals. The gene expression of y^+^LAT1 tended to show higher values in the LPAA animals in the duodenum; no diet-related effect could be identified in the jejunum and no expression in the ileum. The gene expression of B^0^AT_1_ was not influenced by the diet in the duodenum, but a higher expression was shown in the ileum of pigs that were fed the LPAA diet. The expression rates of the animals of the HP diet showed no difference between duodenum and jejunum for b^0,+^AT and for B^0^AT_1_, but the expression in the jejunum was higher compared to the ileum. In this study, the LPAA diets contained free AA, which could be absorbed as soon as they arrived in the small intestine, whereas the HP diet only contained protein-bound AA, which had to be digested by pancreatic proteases [32]. The release and absorption of most protein-bound AA occurs in the jejunum [54]. Consequently, the level of expression of the respective AA transporters in pigs that have a high proportion of protein-bound AA in the feed is expected to be higher in the mucosa of the jejunum than in the duodenum or ileum. In addition, the high level of free lysine in the LPAA diet could stimulate the level of lysine transporters (b^0,+^AT) in the duodenum, in contrast to the animals in the HP diet group [32]. Based on previous studies, it was shown that the expression of AA transporters represented their functional activity; thus, the increase in expression of the lysine transporters in the duodenum could be a strong indicator for an improved absorption capacity of the pigs fed with the LPAA diet [50,55].

The uptake of dietary lysine by enterocytes in the small intestine of pigs is significantly facilitated by the cationic AA transporter b^0,+^AT, which is mainly expressed in the apical membrane of the epithelial cells [50,56]. Expression of b^0,+^AT was demonstrated in the duodenum, jejunum and ileum in Tibetan pigs and growing pigs [47,57]. The system b^0,+^AT acts as an antiporter, which exchanges leucine for lysine, whereby leucine passes into the intestinal lumen (Figure 2) [58]. In the study from Morales et al. (2017), the expression of b^0,+^AT in the duodenum was about twice as high in the LPAA-treated pigs compared to the HP-treated pigs. Similar effects were shown in another study with growing pigs [32,57]. Interestingly, the expression of b^0,+^AT in the jejunum was not influenced by the dietary treatment, which could also be shown in two other studies [59,60]. The presence of free lysine in the duodenal digesta stimulates the expression of b^0,+^AT in the duodenal mucosa [32]. The transport of lysine from the intestinal lumen into the blood begins with b^0,+^AT and ends with y^+^LAT1, which transports lysine through the basolateral membrane of the enterocytes into the blood [50]. This system also acts as an antiporter similar to b^0,+^AT, exchanging lysine from the enterocyte into the blood with leucine [61]. In the study from Morales et al. (2017), the expression of y^+^LAT1 in the duodenum was about 50% higher in the pigs fed the LPAA diet, whereas expression in the jejunum was not affected by the diet. These results indicated a complementary activity in the process of transferring cationic AA from the intestinal lumen to the blood between y^+^LAT1 and b^0,+^AT. The data also show that pigs on the LPAA diet express more y^+^LAT1 in the jejunum than in the duodenum. These results support the hypothesis that the form of the AA (bound or free) in the diet influences the expression of y^+^LAT1. The absorption of most neutral AA in the small intestine is mediated by the system B^0^AT_1_, which is localized exclusively in the apical membrane of the enterocytes and transports all neutral AA, with a clear preference for leucine, isoleucine, valine and methionine [50,62]. In literature it was described that supplementation with branched-chain AA did not influence the expression of B^0^AT_1_ in the jejunum [59]. It was also found that B^0^AT_1_ expression in the duodenum, jejunum and ileum is not changed in growing pigs, regardless of whether they were fed a diet with less protein and supplemented free AA or a diet with a high protein content without supplemented AA [60]. In the study from Morales et al. (2017), no influence of the diet on the B^0^AT_1_ expression in the duodenum and jejunum could be determined, but the expression in the ileum was increased 4.1-fold when the animals were fed with LPAA diet. The authors argued that the likewise high proportion of 80% bound protein in the LPAA diet caused the reduced expression in the duodenum and jejunum. However, it is unclear why the B^0^AT_1_ expression rate was increased in the ileum of the LPAA group. One explanation would be the increased proportion of wheat protein in the LPAA diet and the higher proportion of neutral AA, which are increasingly digested in the distal small intestine [32]. A study in mice found that the expression of the transporter B^0^AT_1_ was the same in all small intestine segments, but in humans there was an increase in expression from the duodenum to the ileum [63,64]. Diet-dependent effects were identified in pigs with higher expression rates in the jejunum compared to duodenum and ileum when pigs were fed with LPAA and HP diet [32], whereas no differences between the porcine intestinal segments were identified when a lysine-rich and a lysine-deficient ratio was fed [60]. These data indicate that there are differences in expression depending on the species and the body weight of the animals [32,60].

The intestinal frequency of AA transporters probably correlates with the absorption and availability of the AA that these transporters prefer to ingest. The AA content in the diet and the form of the AA ingested by the animals are reflected in the serum concentration of free AA [65,66]. The serum concentration of the AA that were supplemented in the LPAA diet were between 90% and 110% higher than in the animals fed the HP diet. A connection between the higher serum concentrations and the higher expression rates of the b^0,+^AT transporters could also be recognized. This indicates that the serum concentration of the AA reflects the different frequencies and activities of the AA transporters [32].

Another factor influencing the expression of transporters could be the genetic potential of the animals. Animals with higher feed efficiency could have a higher expression level of these transporters. A study with pigs examined the gene expression of various nutrient transporters in a population with animals of different feed efficiencies [67]. The authors were able to determine that pigs with a low residual feed intake had an increased monosaccharide transporter expression in the jejunum. An altered expression in the duodenum or ileum could not be determined. The authors assumed that the changed expression in the jejunum but the constant expression in the duodenum and ileum could be explained by the fact that the main digestion of the food takes place in the stomach and duodenum, whereas the absorption of the nutrients takes place predominantly in the jejunum. The authors also assumed that a diet that contains less easily digestible components could also lead to a change in the ileum. These results indicated that animals with a higher efficiency were elevated in nutrient degradation and its subsequent transport [67].

## 4. Investigation of Proteolytic Capacities in the Host Proteome

The use of proteomics has become a successful instrument for an improved understanding of the physiology in the digestive tract of pigs. The typical workflow in proteomics includes protein extraction, protein purification, tryptic digestion of proteins to peptides, separation of the peptides and the tandem mass spectrometry (MS/ MS) analysis. The proteins are then identified by comparing the mass spectra of the experiment with theoretical mass spectra from sequence databases [68,69].

This methodology was applied by Tröscher-Mußotter et al. (2019) to identify pig proteins along the entire gastrointestinal tract of pigs using the *Sus scrofa* sequence database [70]. The body’s own proteins of the mucosa could mainly be assigned to the metabolism and the organismal system of the cells. The dominant cluster within these proteins was assigned to the cellular processes including keratin 8, annexin A2, and albumin as predominant proteins. A relevant amount of hemoglobin beta-bound protein was identified in the stomach samples. It was shown that these proteins decreased across the small intestine and the relative frequency increased again in the large intestine. The general cluster of metabolism was dominated by proteins involved in oxidative phosphorylation, such as the ATP synthesis and the transport proteins. In general, the digesta samples were significantly more heterogeneous than the samples of the mucosa, which may have been caused by using pigs fed with different diets. Due to the better reproducibility and the localization, the mucus layer should be considered to investigate host functions such as the secretion of digestive enzymes and the expression of transport proteins [70].

Stomach, duodenum and jejunum were further investigated by Ma et al. (2018) to examine the influence of the protein content in the feed on the mucosal proteome [71]. Throughout the samples and sections, a mean of 4750 proteins were identified and used to determine the differential expressed (DE) proteins between the three treatments differing in crude protein (CP) content in the feed (14, 17, 20% CP). A minor number of DE proteins were identified, which included ribosome, vitamin and lipid digestion and absorption, fat digestion and absorption, etc., which were up-regulated and phagosomes, cysteine and methionine metabolism, which were down-regulated in gastric mucosa comparing 17% CP vs. 20% CP. In the duodenum, proteins related to carbon metabolism, glycolysis and immune system functions were up-regulated, whereas pancreatic secretion was down-regulated comparing 17% CP vs. 20% CP. The same treatment comparison showed an up-regulation for protein digestion and absorption, complement and coagulation cascades in jejunum mucosal samples [71]. DE proteins comparing 14% CP vs. 20% CP showed a similar trend with up-regulated proteins of lipid metabolism in gastric and duodenal mucosa, and protein digestion, absorption and transport in jejunal mucosa. Conversely, carbon (starch and sucrose), lipid digestion and absorption were down-regulated in duodenum. After several studies that showed the positive impact of lower CP dietary levels on growth parameters and animal health [72,73,74], the data of this study gave a first in-depth insight of the influence of the CP content on the molecular level of metabolic proteins. A median CP level (17%) influences the protein, carbohydrate and lipid digestion and absorption. AA transporters were up-regulated in the median CP treatments, and fat and vitamins uptake seemed to be supported by median CP but not with 14% CP [71].

In a study with rats, it could be shown that animals that were fed a low protein diet showed a normal or even increased absorption capacity for essential AA in the intestine. This indicates that the body starts the specific signal transduction and increases the expression of AA transporters and decreases the AA metabolism to meet the specific needs [75].

## 5. Investigation of Proteolytic Capacities in the Intestinal Microbiome

The gastrointestinal tract of pigs is populated by numerous microorganisms, where bacteria make up the highest proportion. The intestinal microbiome forms a specific ecosystem benefit from the nutrients and the energy that are available from the feed and the host, but also produce substances such as vitamins, organic acids and gases for the host. Thus, microorganisms have a high impact on the health of the host and the microbiome composition can change dynamically due to nutrition, environmental stress and illness [76]. The bacteria in the gastrointestinal tract are quantitatively listed at the phyla level by Firmicutes, Bacteroidetes, Proteobacteria, and Spirochaetes, whereas Fibrobacteres, Actinobacteria, Tenericutes, Synergistetes and Planctomycetes make up less than 1% of all 16S rRNA gene sequences [77,78,79]. The intestinal microbiome can be identified by using 16S rRNA sequencing using DNA extracts from the intestinal sections and specific primers that enable the amplification of a hypervariable region of the 16S rRNA gene. By evaluating the sequencing data, bacteria present in the respective sample material can be identified and the relative abundance is used to compare the influence of dietary strategies and other factors [78,79,80]. In addition, using metatranscriptomics and metaproteomics, information about the active microbes are also available.

Metaproteomics has been used to study microbial proteins from different types of samples in order to identify and quantify in which metabolic pathways they are involved [81]. In addition, the proteomic status of the host as well as feed-proteins can be examined by co-extracting microbial, host and feed proteins [70,82,83]. Thus, the co-extraction of these protein groups enables a comprehensive insight from the same sample. A study that examined the proteome in pigs identified 2917 bacterial proteins in the digesta and 973 bacterial proteins in the mucosa. In addition, a total of 4550 host proteins were identified. In the jejunum and ileum, the bulk of the protein in the digesta was from the host. In addition, over 75% of the proteins in the mucosa were porcine and of non-microbial origin. The main phyla Firmicutes, Bacteroidetes and Actinobacteria were detected in this study [70]. In the study from Gierse et al. (2020), the 16S rRNA gene amplicon and the metaproteomics analysis showed a microbiome dominated by Prevotellaceae, Lactobacillaceae, Lachnospiraceae, Ruminococcaceae and Clostridiaceae [83]. Both studies are in line with several sequencing studies in pigs assigning the highest occurrence to Firmicutes and Bacteroidetes [78,79,84,85]. The protein pathway analysis in Tröscher-Mußotter et al. (2019) identified bacterial proteins involved in energy production, energy conversion, translation, and carbohydrate transport and metabolism. In particular, the proteins involved in the energy metabolism showed similar relative frequencies in the mucosa (23–32%) and the digesta (23–25%) in all animals, which was similar to the results of the study from Haange et al. (2012) analyzing the metaproteome of rats [70,86]. In the mucosa samples, the functions of the proteins could mainly be assigned to the oxidative phosphorylation, carbon metabolism and carbon fixation, whereas the proteins in the digesta samples were additionally assigned to the pyruvate metabolism. In the small intestine, more proteins were identified that are part of carbohydrate metabolism, including glycolysis and gluconeogenesis compared to the large intestine. Proteins related to the biosynthesis of AA occurred with an average of 5% in the digesta and 2% and 9% in the colon and appendix mucosa, respectively. Besides AA biosynthesis, the detection of proteins involved in protein degradation such as peptidases or proteases were of very low abundance along the porcine GIT [70]. The current lack of studies investigating the influence of the protein source or AA supplementation in the feed on the intestinal microbiome refuses any conclusion about the possible interactions for this specific topic. Thus, further research has to be undertaken to study the impact of the intestinal microbiome for protein degradation or AA transformation, and the possible beneficial role of microbes for the host.

## 6. Conclusions and Perspective

The present literature shows the specific activities and expression levels of proteolytic enzymes and AA transporters depending on the availability of free or bound-AA in the feed. It appears that pigs that are fed free AA to a ration with a low protein content make physiological adjustments to adapt the absorption pattern. Above all, the result of the higher occurrence of cationic AA transporters in the duodenum, combined with an increase in the serum concentration of cationic AA in animals on a low protein diet supplemented with free AA, indicate that the absorption capacity increases even if the enzyme activity is reduced [32]. It was also shown that a reduction in the protein content from 20% to 17% or 14%, supplemented with free AA, had a negative impact on the morphology of the small intestine and the pepsin activity. Decreasing blood urea nitrogen and ammonia levels in the colon also showed that nitrogen excretion was reduced. Combined with the observed lower growth performance in piglets from another study, this indicates that a reduced protein content of 3% supplemented with lysine, methionine, threonine and tryptophan corresponds to an alternative feeding recommendation [24,87]. It also suggests that improvements in nutrient digestibility results from changes in nutrient transporter and enzyme gene expression as well as a change in microbial activity [67]. It also became clear that the functional protein clusters between the small and large intestines differ significantly, which is in line with the physiological differences between these two intestinal segments. Differences are also known among the intestinal microbiomes, showing significant variations along the GIT and between lumen and mucosa-associated microbiomes.

Besides the benefits that are obtained by analyzing the (meta-)proteomes of pigs to gain more insight on the host and microbiome related functions, the method also depends on good protein coverage, a constant expansion of sequence databases and proper gene annotations. An alternative is the use of (meta-)transcriptomics, which merge information about presence and expression level of genes by analyzing their sequences. The total of all RNA transcripts occurring in the organism, both coding and non-coding, is recorded. Some research groups have already used the transcriptome level to study feed efficiency in pigs [88,89,90,91,92]. All of these studies showed that the transcriptomic method is a powerful tool to gain a deeper insight into the genetic differences between efficient and less efficient animals. This should be adopted to the specific topic of protein efficiency as related to the identified key genes or gene clusters, which can serve as future breeding targets for an enhanced protein efficiency in pigs.

## Figures and Tables

**Table 1:** Enzymes involved in protein digestion, their products and detection methods.

Enzyme (Source)	Function	Product	Detection	Substrate	Wave Length
**Endopeptidase**					
Pepsin (stomach)	Cleavage peptides with aromatic AA	Peptide (AA)	F	Synthetic peptide substrate	Ex: 328 nm Em: 418 nm
Trypsin (pancreas)	Cleavage peptides with basic AA	Peptide (AA)	A	Synthetic substrate	405 nm
Chymotrypsin (pancreas)	Cleavage peptides with aromatic AA and tryptophan	Peptide (AA)	F	Synthetic fluorogenic substrate	Ex: 380 nm Em: 460 nm
Elastase (pancreas)	Cleavage peptides with neutral AA without ring system	Peptide (AA)	F	Synthetic substrate	Ex: 380 nm Em: 500 nm
**Exopeptidase**					
Carboxypeptidase A (pancreas)	Cleavage peptides with C-terminal AA	AA, Peptide	A	N-(4-methoxyphenylazoformyl)-Phe-OH potassium salt	350 nm
Carboxypeptidase B (pancreas)	Cleavage peptides with C-terminal basic AA	AA, Peptide	A	N-(4-methoxyphenylazoformyl)-Arg-OH HCl	350 nm
Aminopeptidase (BBM)	Cleavage peptides with C-terminal AA	AA, Peptide	F	Fluorogenic substrate	Ex: 384 nm Em: 502 nm
**Other Peptidase**					
y-Glutamyl-transpeptidase (BBM)	Cleavage the AA glutamic acid from the tripeptide glutathione	AA, Dipeptide	A	L γ Glutamyl pNA	418 nm
Dipeptidyl-peptidase (BBM)	Cleavage the AA glutamic acid from the tripeptide glutathione	Dipeptide, Peptide	F	Synthetic substrate	Ex: 360 nm Em: 460 nm

Modified from [17]; BBM, brush border membrane; F, fluorescence; A, absorbance; EX, excitation; EM, emission.

## Data Availability

Not applicable.
